# Supplementary material for: Mcam inhibits macrophage-mediated development of mammary gland through non-canonical Wnt signaling
Source: Nat Commun. 2024 Jan 2;15:36. doi: 10.1038/s41467-023-44338-0 (PMC10761817; doi:10.1038/s41467-023-44338-0)
Supplement: Supplementary file 1 — Supplementary Information [file 41467_2023_44338_MOESM1_ESM.pdf]

Supplementary Information

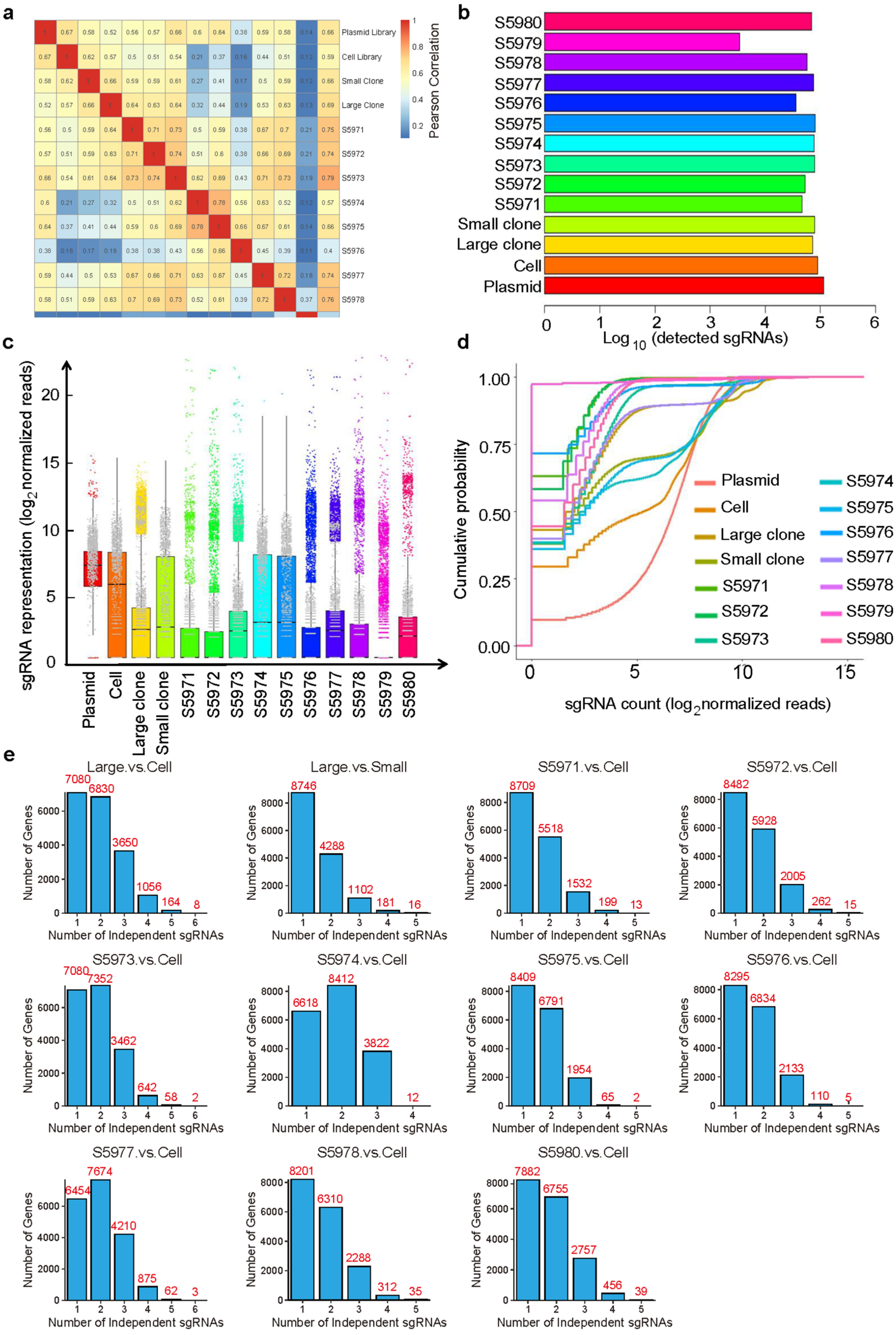

Figure S1. CRISPR/Cas9 screening for MEC regulators using mouse whole-genome GeCKO v2 library.

(a) Pearson correlation coefficients of normalized sgRNA read counts from plasmids of mouse GeCKO v2 library, stably transfected cells, large clones, small clones, and regenerated mammary tissues. n = 8 mice (S5973–S5980) for transplantation. (b) Number of unique sgRNAs in mouse GeCKO v2 plasmid library, stably transfected cells, large clones, small clones, and regenerated mammary tissues as in (a). (c) Boxplot of sgRNA-normalized read counts for mouse GeCKO v2 plasmid library, stably transfected cells, large clones, small clones, and regenerated mammary tissues as in (a). Outliers are shown as colored dots for each respective sample. Gray dots overlaid on each boxplot indicate read counts for 1 000 control (non-targeting) sgRNAs in GeCKO v2 library. (d) Cumulative probability distribution of library sgRNAs in mouse GeCKO v2 plasmid library, stably transfected cells, large clones, small clones, and regenerated mammary tissues as in (a). (e) The number of genes with various significantly enriched sgRNAs targeting those genes in each sample.

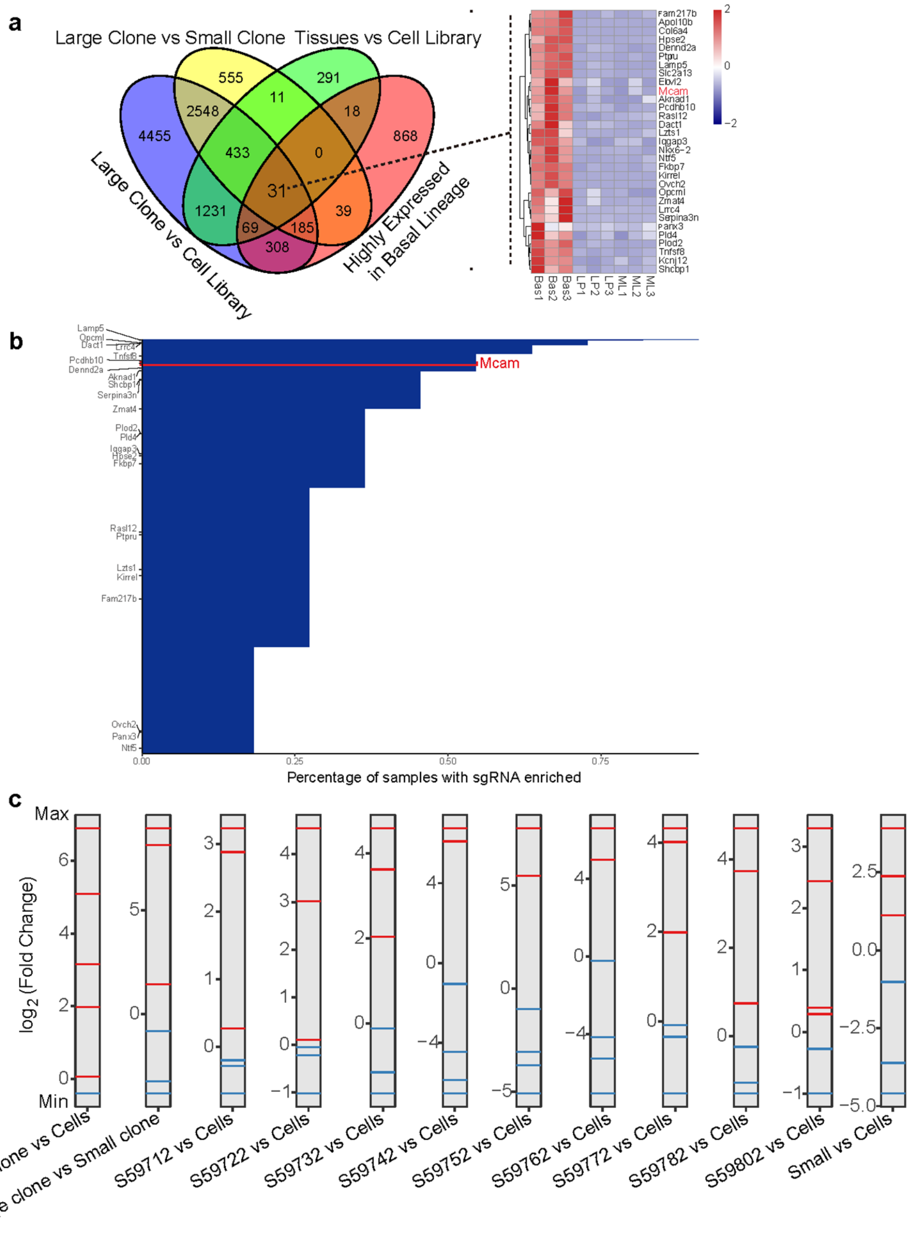

Figure S2. Screening of Mcam based on various methods.

(a) Overlap analysis of top enriched sgRNAs in large clones and regenerated mammary tissues, which the targeted genes were also highly expressed in basal cells than luminal cells. (b) Candidate genes targeted by two or more sgRNAs in large clones and regenerated mammary tissues. (c) The fold changes of each *Mcam* sgRNA between various sample pairs. The red lines represent enriched individual sgRNAs

between various comparisons, and the blue ones represent non-enriched sgRNAs.

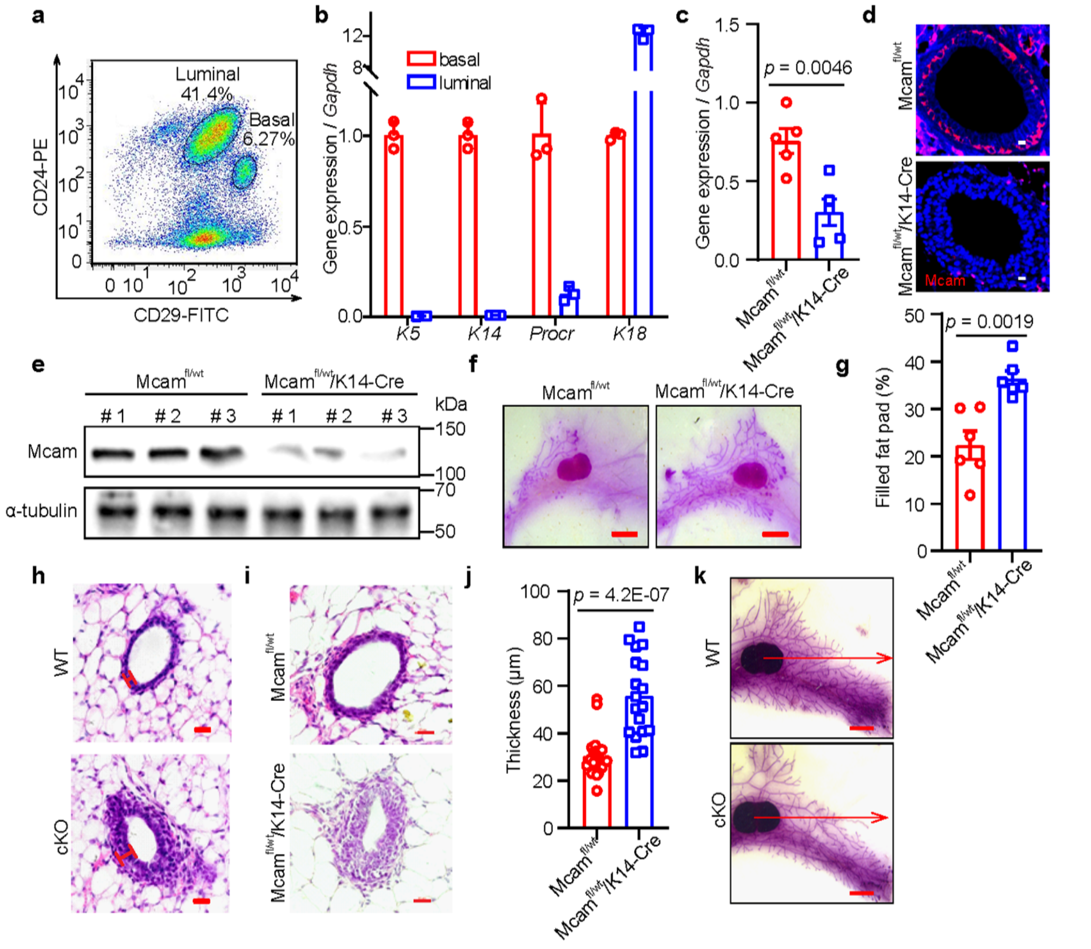

Figure S3. Mcam loss by K14-Cre mice promotes mammary ductal elongation.

(a) Mammary basal and luminal cells sorted by FACS from 8-week-old nulliparous mammary glands. (b) qRT-PCR analysis of basal markers (*K5*, *K14*, and *Procr*) and luminal marker (*K18*). n = 3 biological replicates. (c) qRT-PCR analysis of *Mcam* mRNA expression in MECs from Mcam$^{fl/wt}$ and Mcam$^{fl/wt}$/K14-Cre mice. n = 5 mice per genotype. (d) IF staining showing knockout efficiency of Mcam in Mcam$^{fl/wt}$ and Mcam$^{fl/wt}$/K14-Cre mice. Scale bar, 5 μm. (e) Western blotting showing Mcam protein expression in Mcam$^{fl/wt}$ and Mcam$^{fl/wt}$/K14-Cre MECs. n = 3 mice for each group. (f-g) Representative images (f) and their quantification (g) of mammary duct

extension of whole-mount staining in Mcam$^{fl/wt}$ and Mcam$^{fl/wt}$/K14-Cre mice at 5 weeks of age. Scale bar, 5 mm. n = 6 mice for each group. (h) Schematic diagram of measurement method in H&E staining. Scale bar, 20 μm. Red symbol indicates the measured thickness. (i) Representative images of mammary tissues in 5-week-old Mcam$^{fl/wt}$ and Mcam$^{fl/wt}$/K14-Cre mice by H&E staining. Scale bar, 20 μm. (j) Quantification of mammary duct thickness in Mcam$^{fl/wt}$ and Mcam$^{fl/wt}$/K14-Cre mice. n = 22 ducts in Mcam$^{fl/wt}$ mice, n = 18 ducts in Mcam$^{fl/wt}$/K14-cre. (k) Representative images of whole-mount staining in WT and cKO (depleted by MMTV-Cre mice) mice at 8 weeks of age. Scale bar, 5 mm. Data are means ± SEM. Two-sided Student's *t* test was used to evaluate statistical significance. Source data are provided as a Source Data file.

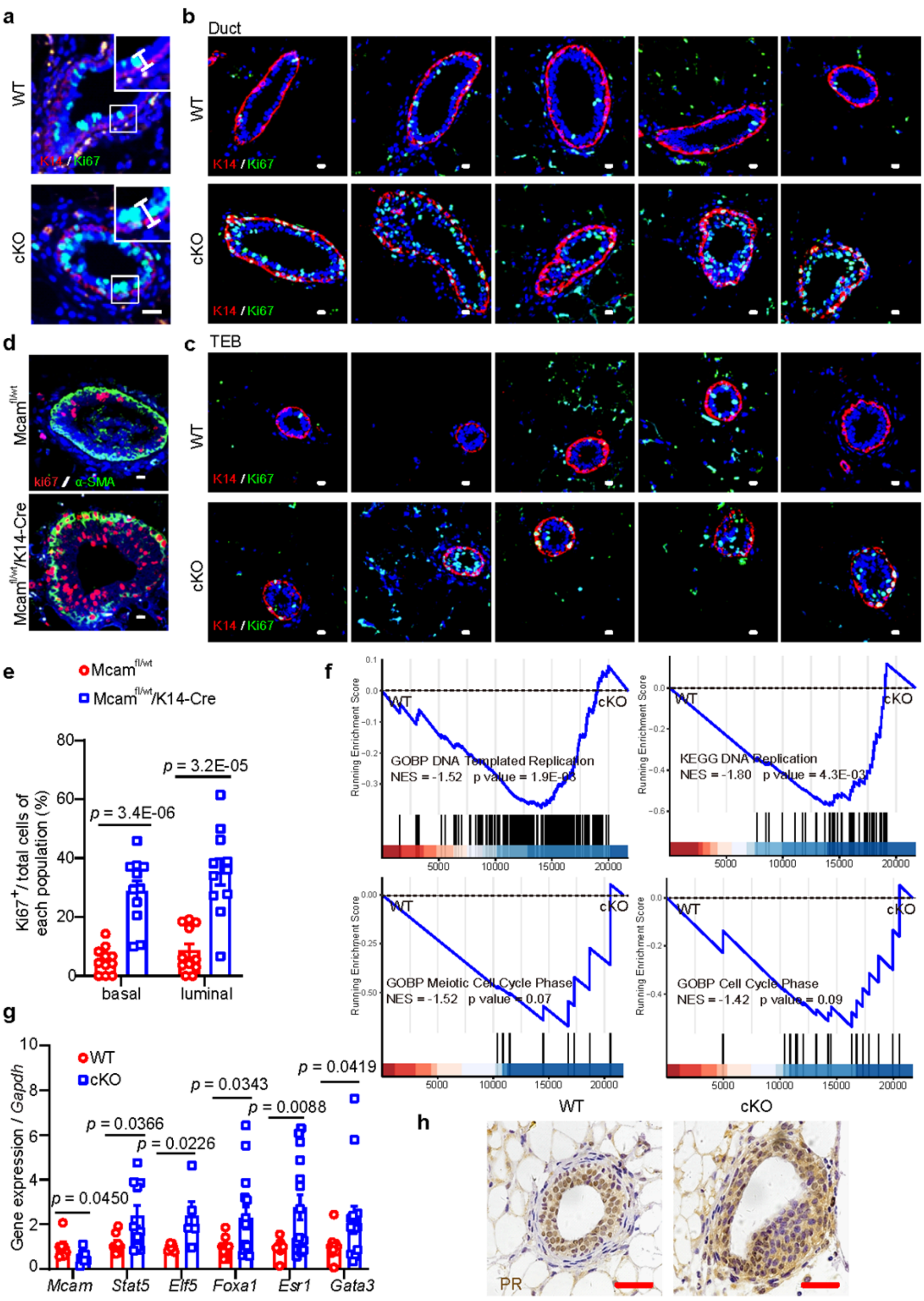

Figure S4. Mcam loss promotes the proliferation and differentiation of MECs.

(a) Schematic diagram of measurement methods in IF staining. Scale bar, 10 μm. White symbol indicates the measured thickness. (b-c) Immunostaining of K14 (red)

and Ki67 (green) expression in ductal (b) and TEB (c) structure of WT and cKO mice by MMTV-Cre. Scale bar, 10 μm. (d) Immunostaining of α-SMA (red) and Ki67 (green) expression in $Mcam^{fl/wt}$ and $Mcam^{fl/wt}$/K14-Cre mice. Scale bar, 10 μm. (e) $Ki67^{+}$ cells in basal and luminal cell populations of $Mcam^{fl/wt}$ and $Mcam^{fl/wt}$/K14-Cre mice. n = 11 sections in each group. (f) GSEA of DNA replication and cell cycle pathways in cKO mice compared with WT mice. NES, normalized enrichment score. (g) qRT-PCR analysis of *Mcam* and various differentiation genes at mRNA level in MECs from WT and cKO mice by MMTV-Cre. 7-12 individual WT mice and 5-15 individual cKO mice were taken for analysis, and each dot represents the average of three replicates for a single mouse. (h) IHC staining of progesterone receptor (PR) in WT and cKO mammary tissues by MMTV-Cre. Scale bar, 20 μm. Data are means ± SEM. Two-sided Student's *t* test was used to evaluate statistical significance. Source data are provided as a Source Data file.

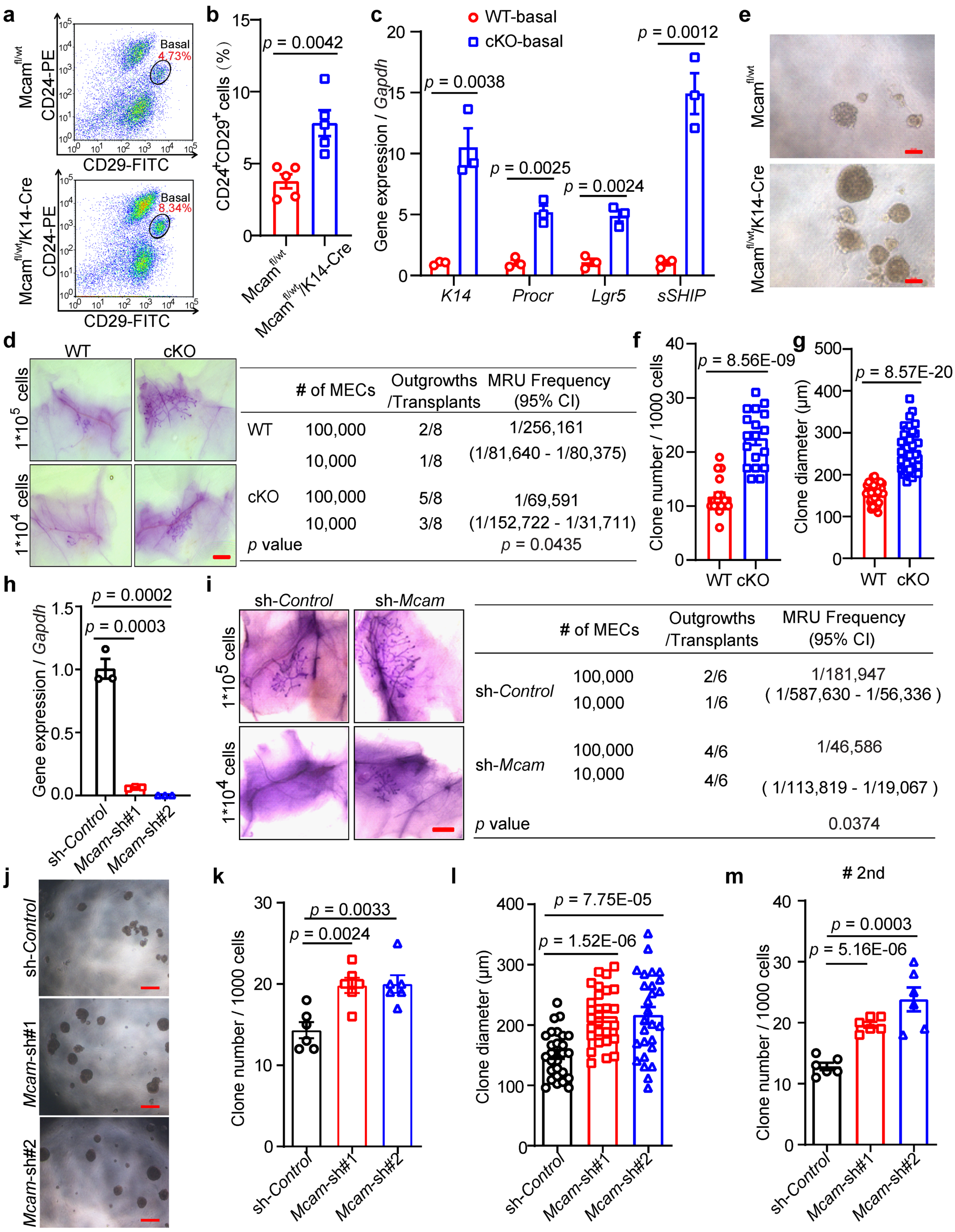

Figure S5. Loss of Mcam promotes regenerative capacity.

(a-b) FACS analysis (a) and statistical ratios (b) of basal cell population ($Lin^{-}CD24^{+}CD29^{+}$) in $Mcam^{fl/wt}$ and $Mcam^{fl/wt}$/K14-Cre mice. 5 individual mice for each genotype. (c) qRT-PCR results for indicated genes in basal cells isolated from

mammary tissues of Mcam WT or cKO mice by MMTV-Cre. n = 3 biological replicates in each group. (d) Representative images (left) and the reconstitution efficiency at limiting dilution (right) of whole-mount-stained mammary outgrowths derived from transplantation of WT and cKO primary MECs and harvested at 5 weeks after transplantation. Scale bar, 5 mm. n = 8 in each group. (e-g) Representative images (e) and statistical results of clone numbers (f, n = 18), clone diameters (g, n = 40) of colonies formed by WT and heterozygous cKO (depleted by K14-Cre mice) MECs, respectively. Scale bar, 100 μm. (h) qRT-PCR results for *Mcam* in MECs treated with *Mcam* shRNAs, and *control* shRNAs (negative control). n = 3 biological replicates. (i) Representative images (left) and the reconstitution efficiency at limiting dilution (right) of whole-mount-stained mammary outgrowths derived from transplantation of sh-*Control*- or sh-*Mcam*-lentivirally transduced MECs and harvested at 5 weeks after transplantation. Scale bar, 5 mm. n = 6 mice in each group. (j-m) Representative images (k) and statistical results of clone numbers (k, n = 6), clone diameters (l, n = 27), and clone numbers at 2nd passage (m, n = 6) of colonies formed by MECs with indicated lentivirus treatment, respectively. Scale bar, 500 μm. Data are means ± SEM. Two-sided Student's *t* test was used to evaluate statistical significance. Source data are provided as a Source Data file.

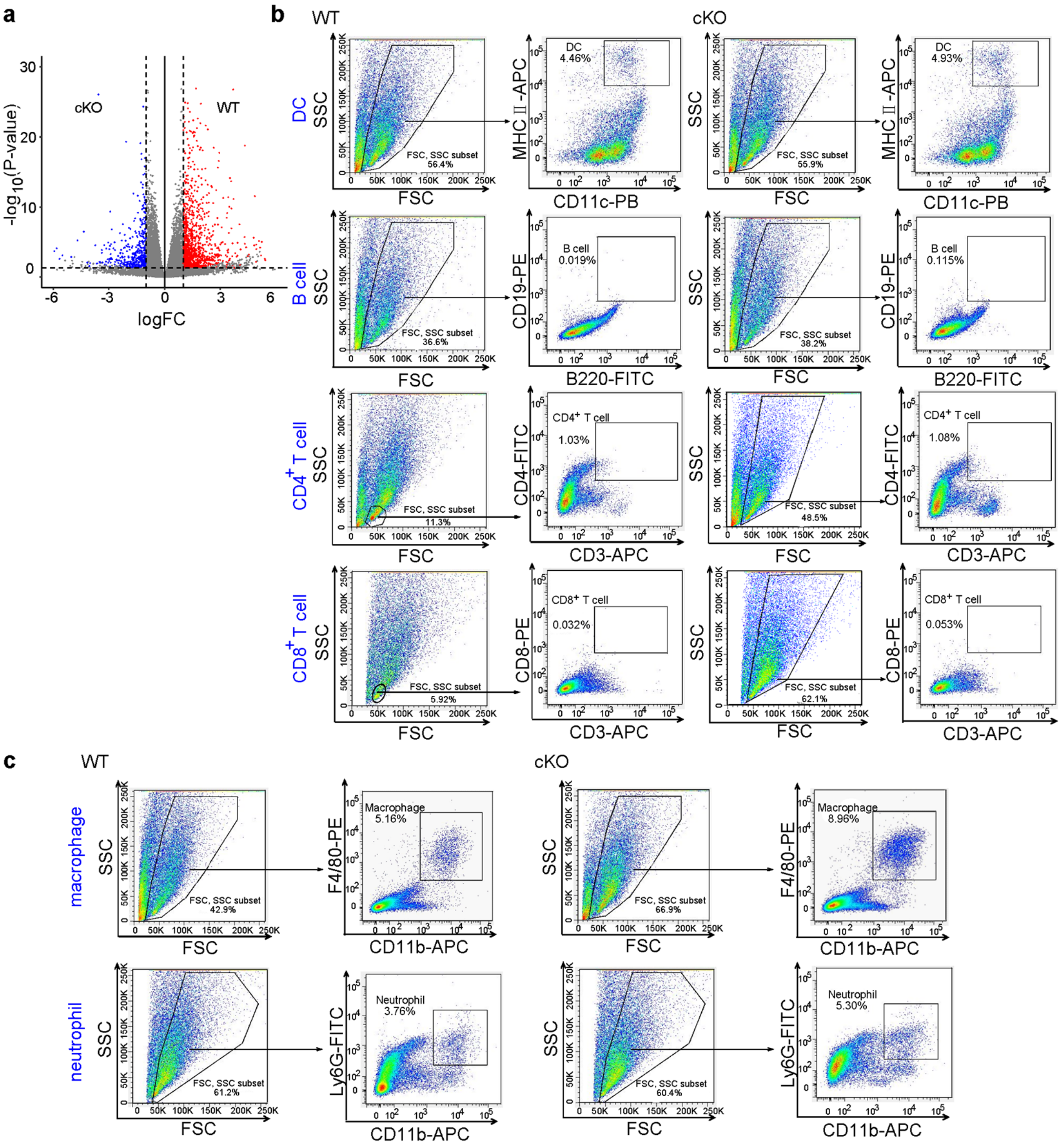

Figure S6. Loss of Mcam increases the proportion of macrophages.

(a) Volcano plot showing DEGs in mammary tissues of WT and cKO mice. (b) The gating strategy of FACS analysis for indicated cell population in mammary tissues of WT and cKO mice by MMTV-Cre. (c) The gating strategy of FACS analysis for macrophage and neutrophil in mammary tissues of WT and cKO mice by MMTV-Cre.

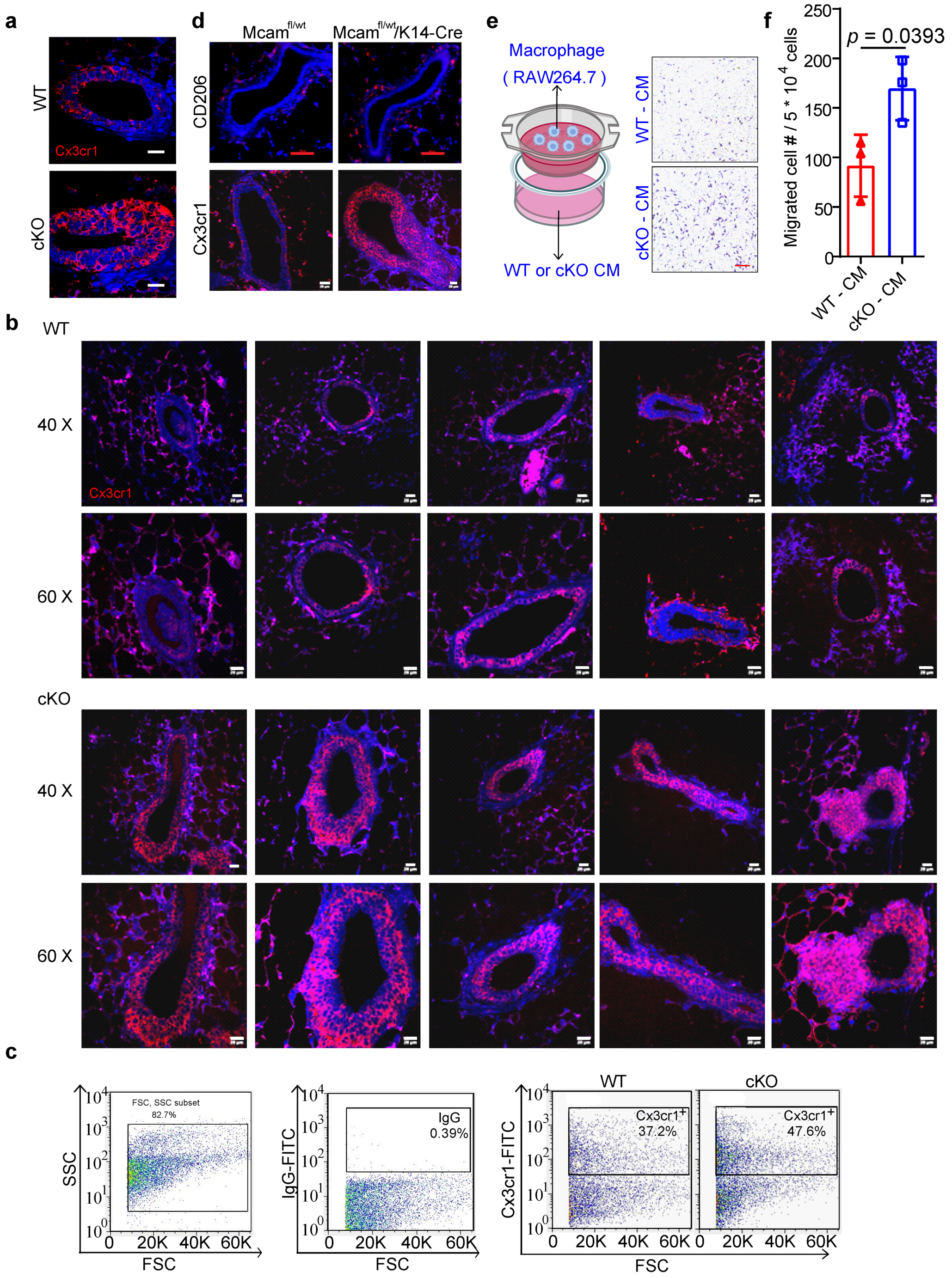

Figure S7. Loss of Mcam promotes macrophages recruitment.

(a-b) IF staining of Cx3cr1 with different magnification in mammary tissues of WT and cKO mice by MMTV-Cre. Scale bar, 20 μm. (c) The gating strategy of FACS analysis for Cx3cr1^{+} macrophages in mammary tissues of WT and cKO mice. (d) IF

images of $CD206^+$ and $Cx3cr1^+$ macrophages in $Mcam^{fl/wt}$ and $Mcam^{fl/wt}$/K14-Cre mice. Scale bar, 50 μm for CD206 staining, and 20 μm for Cx3cr1 staining. (e-f) Schematic of co-culture assay and representative images (e) (The left panel was created with BioRender.com.), and quantification (f) of migrating macrophages after WT and cKO CM treatment. Macrophage cell line in upper chamber were co-cultured with CM from WT and cKO primary MECs in lower chamber. n = 3 biological replicates. Scale bar, 200 μm. Data are means ± SEM. Two-sided Student's *t* test was used to evaluate statistical significance. Source data are provided as a Source Data file.

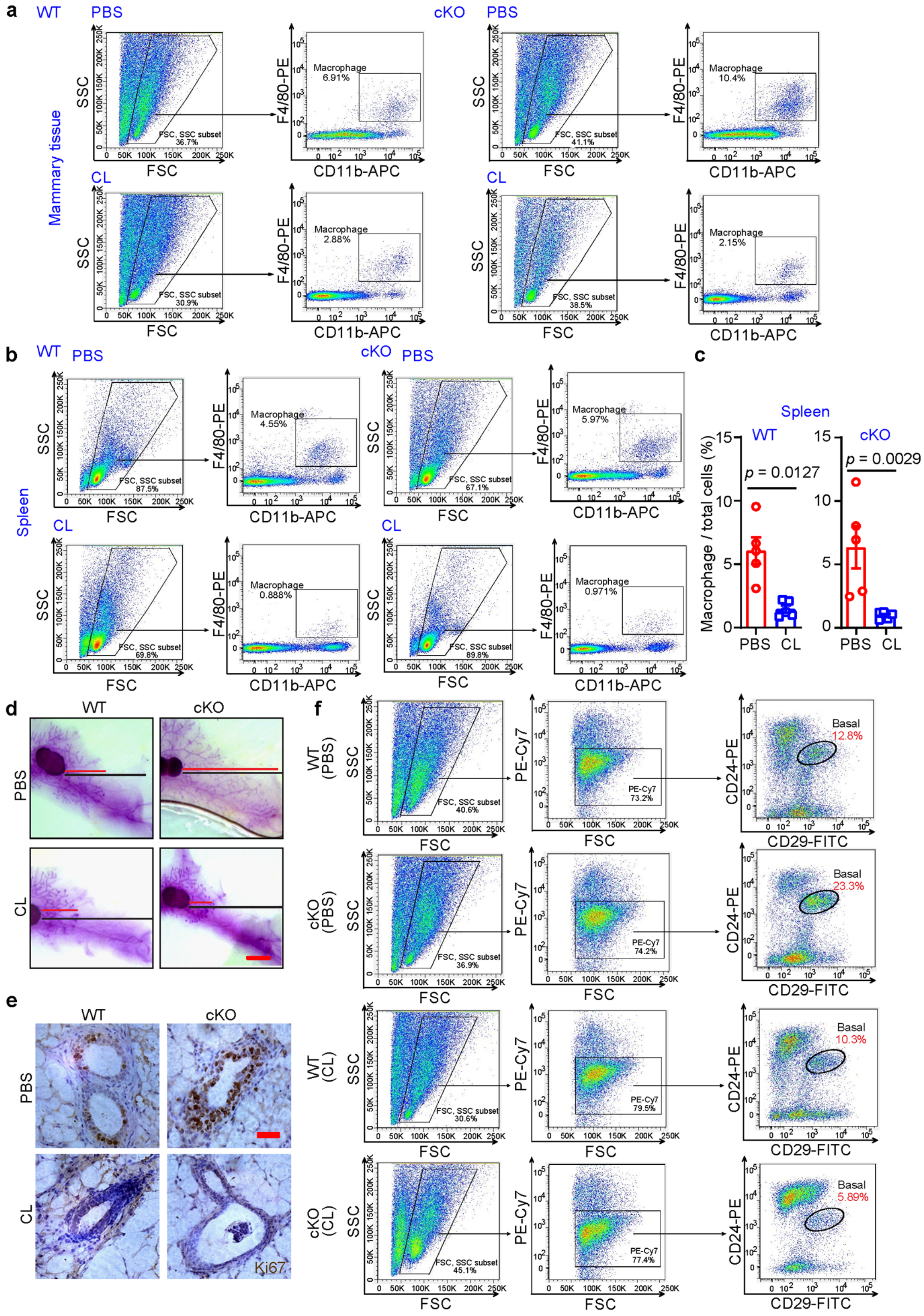

Figure S8. Depletion of macrophages reduces regenerative capacity of MECs upon Mcam loss by MMTV-Cre.

(a-b) The gating strategy of FACS analysis for F4/80^{+}CD11b^{+} macrophages in

mammary tissues (a) or spleen (b) of WT and cKO mice treated with or without CL. (c) Quantification of panel b. n = 5 mice in each group. (d) Statistical method of the proportion of mammary duct filling fat pad. Red line, the length of the mammary duct. Black line, the length of the fat pad. Scale bar, 5 mm. (e) IHC images of Ki67 in WT and cKO mice with or without CL treatment. Scale bar, 20 μm. (f) The gating strategy of FACS analysis of basal population in WT and cKO mice treated with or without CL. Data are means ± SEM. Two-sided Student's *t* test was used to evaluate statistical significance. Source data are provided as a Source Data file.

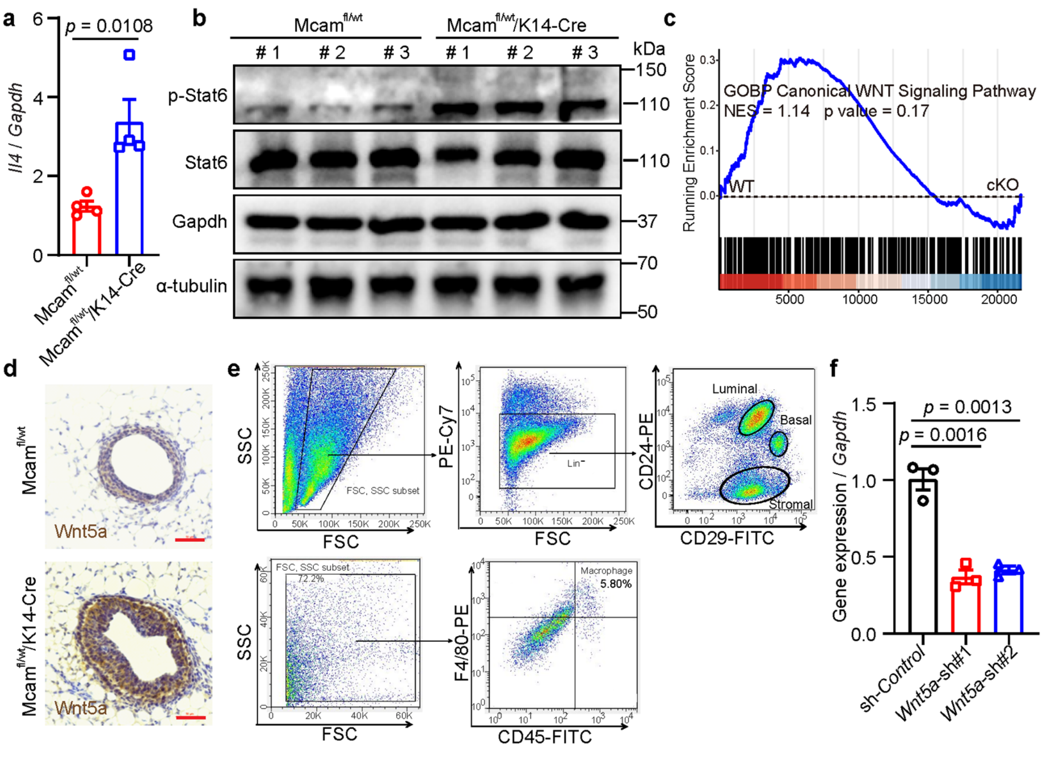

Figure S9. Mcam loss activates Il4-Stat6 pathway and increases Wnt5a expression.

(a) qRT-PCR analysis of expression levels of *Il4* in MECs from Mcam$^{fl/wt}$ and Mcam$^{fl/wt}$/K14-Cre mice. n = 4 biological replicates in each group. (b) Western blotting showing Stat6 and p-Stat6 levels in Mcam$^{fl/wt}$ and Mcam$^{fl/wt}$/K14-Cre mammary tissues. 3 independent mice in each groups. (c) GSEA of non-significant enrichment of canonical Wnt signaling signatures between WT and cKO mice. NES, normalized enrichment score. (d) IHC staining of Wnt5a in Mcam$^{fl/wt}$ and Mcam$^{fl/wt}$/K14-Cre mice. Scale bar, 50 μm. (e) The gating strategy of FACS analysis of Lin$^-$ cell, basal cell, luminal cell, stromal cell, and CD45$^+$F4/80$^+$ cell population. (f) Knockdown efficiency of sh*Wnt5a* in isolated macrophages. n = 3 biological

replicates. Data are means ± SEM. Two-sided Student's *t* test was used to evaluate statistical significance. Source data are provided as a Source Data file.

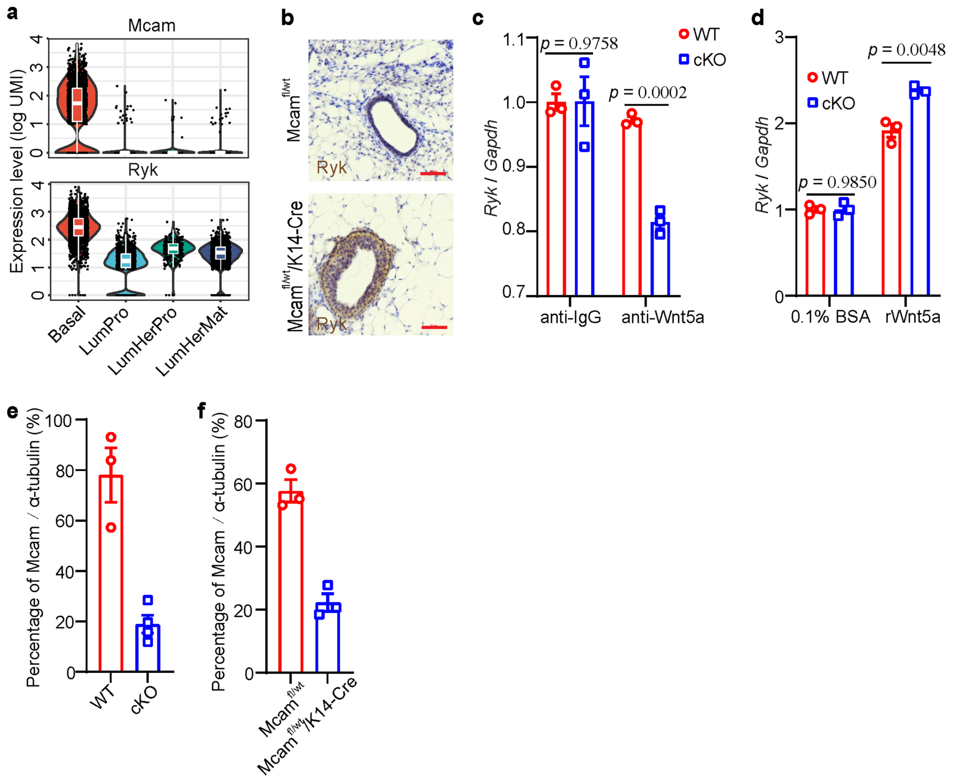

Figure S10. Validation of expression pattern of Mcam and Ryk.

(a) Expression patterns of Mcam and Ryk in various populations according to MEC single-cell sequencing data. (b) IHC staining of Ryk in Mcam$^{fl/wt}$ and Mcam$^{fl/wt}$/K14-Cre mice. Scale bar, 50 µm. (c-d) *Ryk* expression levels in WT and cKO (depleted by MMTV-Cre mice) primary MECs after blocking by Wnt5a antibody (c) or activation by recombinant protein (rWnt5a) (d). n = 3 biological replicates. (e) Percentages of Mcam protein levels to α-tubulin (left) and their raw gray intensities (right) of Figure 1h. n = 3 mice in WT group, and n = 4 mice in cKO group. (f) Percentages of Mcam protein levels to α-tubulin (left) and their raw gray intensities (right) of Figure S3e. n = 3 mice in each group. Data are means ± SEM. Two-sided Student's *t* test was used to evaluate statistical significance. Source data

are provided as a Source Data file.
