# Supplementary material for: Mcam inhibits macrophage-mediated development of mammary gland through non-canonical Wnt signaling
Source: Nat Commun. 2024 Jan 2;15:36. doi: 10.1038/s41467-023-44338-0 (PMC10761817; doi:10.1038/s41467-023-44338-0)
Supplement: Supplementary file 3 — Description of Additional Supplementary Files [file 41467_2023_44338_MOESM3_ESM.pdf]

Description of Additional Supplementary Files

File Name: Supplementary Data 1
Description: The frequency of sgRNAs targeting a gene in each sample

File Name: Supplementary Data 2
Description: GO categories for DEGs

File Name: Supplementary Data 3
Description: Primer sequences for shRNA, quantitative RT-PCR (qRT-PCR), genotyping, and sgRNA readout with barcode

File Name: Supplementary Data 4
Description: Antibodies used for immumofluorescence (IF), immunohistochemistry (IHC), western blot (WB) and blocking experiments

File Name: Supplementary Data 5
Description: Antibodies used for flow cytometry
