# Supplementary material for: Mcam inhibits macrophage-mediated development of mammary gland through non-canonical Wnt signaling
Source: Nat Commun. 2024 Jan 2;15:36. doi: 10.1038/s41467-023-44338-0 (PMC10761817; doi:10.1038/s41467-023-44338-0)
Supplement: Supplementary file 7 — Supplementary Data 4 [file 41467_2023_44338_MOESM7_ESM.doc]

Table S4: Antibodies used for immumofluorescence (IF), immunohistochemistry (IHC), western blot (WB) and blocking experiments

| Antibodies | IF | IHC | WB | Blocking | Company and catalogue number |
| --- | --- | --- | --- | --- | --- |
| CD146-Rb | - | - | 1:500 | - | Abcam; Cat # ab75769  (clone EPR3208) |
| MCAM-Rb | 1:100 | - | - | - | CST; Cat # 81701 |
| K14-Ms | 1:50 | - | - | - | Abcam; Cat # ab49806  (clone LL002) |
| K18-Ms | 1:50 | - | - | - | Abcam; Cat # ab668  (clone C-04) |
| Ki67-Rb | 1:500 | 1:500 | - | - | Abcam; Cat # ab15580 |
| CD206-Rb | 1:1000 | - | - | - | Abcam; Cat # ab64693 |
| Cx3cr1-Rb | 1:50 | - | - | - | Abclonal; Cat # A2890 |
| α-SMA-Ms | 1:200 | - | - | - | Abcam; Cat # ab7817  (clone 1A4) |
| Wnt5a-Rb | - | 1:500 | - | 1:200 | Abcam; Cat # ab235966 |
| Wnt5a-Rb | 1:50 | - | - | - | Abclonal; Cat # A12744 |
| Ryk-Rb | 1:10 | 1:50 | - | 0.5mg/ml | Abcepta; Cat # AP7677a |
| Ryk-Sheep | 1:10 | - | - | - | R&D Systems; Cat # AF4649 |
| InVivoPlus anti-mouse IL4 | - | - | - | 10μg in cell; 100μg in animal | BioXcell; Cat # BP0045-5MG  (clone 11B11) |
| InVivoPlus rat IgG1 isotype control (anti-HRP) | - | - | - | 10μg in cell; 100μg in animal | BioXcell; Cat # BP0088-5MG  (clone HRPN) |
| STAT6-Rb | - | - | 1:1000 | - | CST; Cat # 5397S  (clone D3H4) |
| p-STAT6-Rb | - | - | 1:1000 | - | CST; Cat # 9361S |
| α- tubulin-Ms | - | - | 1:5000 | - | Sigma; Cat # T5168 |
| Gapdh-Rb | - | - | 1:5000 | - | Bioworld; Cat # AP0063 |
| Fluorescein-Labeled Antibody To Rabbit IgG (H+L) | 1:2000 | - | - | - | KPL; Cat # 02-15-06 |
| Fluorescein-Labeled Antibody To Mouse IgG (H+L) | 1:2000 | - | - | - | KPL; Cat # 02-18-06 |
| Cy3 goat anti-mouse | 1:2000 | - | - | - | Life Technologies; Cat # A10521 |
| Cy3 goat anti-rabbit | 1:2000 | - | - | - | Life Technologies; Cat # A10520 |
| DAPI | - | - | - | - | Vector Laboratories; Cat # H-1200 |
| 594 donkey anti-sheep IgG (H+L) | 1:1000 | - | - | - | Thermo Fisher; Cat # A-11016 |
| 488 donkey anti-RAT IgG (H+L) | 1:1000 |  |  |  | Abcam; Cat # ab150153 |
| IgG-Ms | 1:5000 | 1:200 | - | - | Sigma; Cat # A4416 |
| IgG-Rb | 1:5000 | 1:200 | - | - | Sigma; Cat # A6154 |
