# Supplementary material for: Mcam inhibits macrophage-mediated development of mammary gland through non-canonical Wnt signaling
Source: Nat Commun. 2024 Jan 2;15:36. doi: 10.1038/s41467-023-44338-0 (PMC10761817; doi:10.1038/s41467-023-44338-0)
Supplement: Supplementary file 8 — Supplementary Data 5 [file 41467_2023_44338_MOESM8_ESM.doc]

Table S5: Antibodies used for flow cytometry

| Antibody | Catalog # | Company |
| --- | --- | --- |
| PE Rat Anti-Mouse CD24 | 553262 (clone M1/69) | BD Biosciences |
| FITC Hamster Anti-Rat CD29 | 555005 (clone Ha2/5) | BD Biosciences |
| Biotin Rabbit Anti-Mouse TER-119 | 553672 (clone TER-119) | BD Biosciences |
| Biotin Rabbit Anti-Mouse CD31 | 553371 (clone MEC 13.3) | BD Biosciences |
| Biotin Rat Anti-Mouse CD45R/B220 | 553086 (clone RA3-6B2) | BD Biosciences |
| Purified Rat Anti-Mouse CD16/CD32 | 553142 (clone 2.4G2) | BD Biosciences |
| Streptavidin-PE-Cy7 | 557598 | BD Biosciences |
| APC Anti-Mouse CD45.2 | 109813 (clone 104) | Biolegend |
| FITC Anti-Mouse CD45.2 | 109805 (clone 104) | Biolegend |
| PE Anti-Mouse CD45.2 | 109807 (clone 104) | Biolegend |
| Pactific Blue^TM^ Anti-Mouse CD45.2 | 109819 (clone 104) | Biolegend |
| APC Anti-Mouse/Human CD11b | 101211 (clone M1/70) | BioLegend |
| PE Anti-Mouse F4/80 | 123109 (clone BM8) | BioLegend |
| APC Anti-Mouse Ly-6G/Ly-6C (Gr-1) | 108412 (clone RB6-8C5) | Biolegend |
| FITC Anti-Mouse CD4 | 100509 (clone RM4-5) | BioLegend |
| APC Anti-Mouse CD3ε Antibody | 100311 (clone 145-2C11) | Biolegend |
| PE Anti-Mouse CD8a | 100707 (clone 53-6.7) | BioLegend |
| PE Anti-Mouse CD19 | 115507 (clone 6D5) | BioLegend |
| FITC Anti-Mouse/Human CD45R/B220 | 103205 (clone RA3-6B2) | BioLegend |
| Pacific Blue™ Anti-Mouse CD11c | 117322 (clone N418) | Biolegend |
| APC Anti-Mouse I-A/I-E (MHC II) | 107613 (clone M5/114.15.2) | BioLegend |
| CD206-Rb | ab64693 | Abcam |
| Cx3cr1-Rb | ab8021 | Abcam |
| F4/80-RAT | MCA497G (clone Cl:A3-1) | Bio-RAD |
| 488 donkey anti-RAT IgG (H+L) | Ab150153 | Abcam |
| HRP, Goat anti-Rabbit IgG | A6154 | Sigma |
| HRP, Goat anti-RAT IgG | A21040 | Abbkine |
