# Supplementary material for: Mcam inhibits macrophage-mediated development of mammary gland through non-canonical Wnt signaling
Source: Nat Commun. 2024 Jan 2;15:36. doi: 10.1038/s41467-023-44338-0 (PMC10761817; doi:10.1038/s41467-023-44338-0)
Supplement: Supplementary file 9 — Reporting Summary [file 41467_2023_44338_MOESM9_ESM.pdf]

nature portfolio

Corresponding author(s): Baowei Jiao

Last updated by author(s): Dec 4, 2023

Reporting Summary

Nature Portfolio wishes to improve the reproducibility of the work that we publish. This form provides structure for consistency and transparency in reporting. For further information on Nature Portfolio policies, see our Editorial Policies and the Editorial Policy Checklist.

Statistics

For all statistical analyses, confirm that the following items are present in the figure legend, table legend, main text, or Methods section.

n/a	Confirmed	
☐	☒	The exact sample size (*n*) for each experimental group/condition, given as a discrete number and unit of measurement
☐	☒	A statement on whether measurements were taken from distinct samples or whether the same sample was measured repeatedly
☐	☒	The statistical test(s) used AND whether they are one- or two-sided<br>*Only common tests should be described solely by name; describe more complex techniques in the Methods section.*
☒	☐	A description of all covariates tested
☒	☐	A description of any assumptions or corrections, such as tests of normality and adjustment for multiple comparisons
☒	☐	A full description of the statistical parameters including central tendency (e.g. means) or other basic estimates (e.g. regression coefficient) AND variation (e.g. standard deviation) or associated estimates of uncertainty (e.g. confidence intervals)
☐	☒	For null hypothesis testing, the test statistic (e.g. *F*, *t*, *r*) with confidence intervals, effect sizes, degrees of freedom and *P* value noted<br>*Give P values as exact values whenever suitable.*
☒	☐	For Bayesian analysis, information on the choice of priors and Markov chain Monte Carlo settings
☒	☐	For hierarchical and complex designs, identification of the appropriate level for tests and full reporting of outcomes
☒	☐	Estimates of effect sizes (e.g. Cohen's *d*, Pearson's *r*), indicating how they were calculated

Our web collection on statistics for biologists contains articles on many of the points above.

Software and code

Policy information about availability of computer code

Data collection	Microscopy images were taken using NIS-Elements F 4.0. qRT-PCR data were collected using QuantStudio 3. Western blot images were taken using SageCapture software. The raw sequence data reported in this paper have been deposited in the Genome Sequence Archive61 in National Genomics Data Center62, China National Center for Bioinformation / Beijing Institute of Genomics, Chinese Academy of Sciences that are publicly accessible at https://ngdc.cncb.ac.cn/gsa/. The accession codes were: GSA: CRA011830 (https://bigd.big.ac.cn/gsa/browse/CRA011830), GSA: CRA011840 (https://bigd.big.ac.cn/gsa/browse/CRA011840).
Data analysis	Graphs and statistical analyses were performed using GraphPad Prism 6. STAR software STAR_2.4.2a (Dobin A et al., 2013), FeatureCounts v1.4.6-p5 (Liao Y et al., 2014) and edgeR package 1.6 (Robinson MD et al., 2014) were used for RNAseq analysis which is briefly described in the manuscript (Methods). Microscopy images were analyzed using Image-Pro Plus 5.

For manuscripts utilizing custom algorithms or software that are central to the research but not yet described in published literature, software must be made available to editors and reviewers. We strongly encourage code deposition in a community repository (e.g. GitHub). See the Nature Portfolio guidelines for submitting code & software for further information.

Data

Policy information about availability of data

All manuscripts must include a data availability statement. This statement should provide the following information, where applicable:

- Accession codes, unique identifiers, or web links for publicly available datasets
- A description of any restrictions on data availability
- For clinical datasets or third party data, please ensure that the statement adheres to our policy

The raw sequence data generated in this study have been deposited in the Genome Sequence Archive (Chen et al., 2021) in National Genomics Data Center (Members and Partner, 2022), China National Center for Bioinformation / Beijing Institute of Genomics, Chinese Academy of Sciences that are publicly accessible at https://ngdc.cncb.ac.cn/gsa/. The accession codes were: GSA: CRA011830 (https://bigd.big.ac.cn/gsa/browse/CRA011830), GSA: CRA011840 (https://bigd.big.ac.cn/gsa/browse/CRA011840).

Research involving human participants, their data, or biological material

Policy information about studies with human participants or human data. See also policy information about sex, gender (identity/presentation), and sexual orientation and race, ethnicity and racism.

Reporting on sex and gender	N/A
Reporting on race, ethnicity, or other socially relevant groupings	N/A
Population characteristics	N/A
Recruitment	N/A
Ethics oversight	N/A

Note that full information on the approval of the study protocol must also be provided in the manuscript.

Field-specific reporting

Please select the one below that is the best fit for your research. If you are not sure, read the appropriate sections before making your selection.

☒ Life sciences ☐ Behavioural & social sciences ☐ Ecological, evolutionary & environmental sciences

For a reference copy of the document with all sections, see nature.com/documents/nr-reporting-summary-flat.pdf

Life sciences study design

All studies must disclose on these points even when the disclosure is negative.

Sample size	The sample size in each experiment was determined to give an appropriate power of the test.<br>The sample sizes of different experiments, including statistics of pups weight and immunofluorescence microscopy, were chosen accord ing to the previous studies published by others (Zhao et al., 2020).
Data exclusions	No data were excluded.
Replication	All attempts at replication were successful as determined using a statistical analysis.<br>A minimum of 3 individuals (biological replicate) have been processed to perform animal experiments in a minimum of 3 independent experiments (technical replicate) in order to reach statistical significance.<br>All experiments were reliably reproduced.
Randomization	Sample were allocated randomly to experimental groups, except when purpose of the experiment compare difference between control and Mcam KO mice.
Blinding	All experiments were conducted in a double blinded fashion in which the researchers were blinded to group allocation.

Reporting for specific materials, systems and methods

We require information from authors about some types of materials, experimental systems and methods used in many studies. Here, indicate whether each material, system or method listed is relevant to your study. If you are not sure if a list item applies to your research, read the appropriate section before selecting a response.

Materials & experimental systems

n/a	Involved in the study
☐	☒ Antibodies
☐	☒ Eukaryotic cell lines
☒	☐ Palaeontology and archaeology
☐	☒ Animals and other organisms
☒	☐ Clinical data
☒	☐ Dual use research of concern
☒	☐ Plants

Methods

n/a	Involved in the study
☒	☐ ChIP-seq
☐	☒ Flow cytometry
☒	☐ MRI-based neuroimaging

Antibodies

Antibodies used

For immunofluorescence staining, immunohistochemistry, western blot and blocking experiments, antibodies were used as follows: CD146 (clone EPR3208, Abcam,ab75769), MCAM (CST, 81701), K14 (clone LL002, Abcam, ab49806), K18 (clone C-04, Abcam, ab668), Ki67 (Abcam, ab15580), CD206 (Abcam, ab64693), Cx3cr1(Abclonal, A2890), α-SMA(clone 1A4, Abcam, ab7817), Wnt5a (Abcam, ab235966), Wnt5a(Abclonal, A12744), Ryk (Abcepta, AP7677a), Ryk(R&D Systems, AF4649), InVivoPlus anti-mouse IL4(clone 11B11, BioXcell, BP0045-5MG), InVivoPlus rat IgG1 isotype control (anti-HRP) (clone HRPN, BioXcell, BP0088-5MG), STAT6(clone D3H4, CST, 5397S), p-STAT6(CST, 9361S), α- tubulin(Sigma, T5168), Gapdh(Bioworld, AP0063). The secondary antibodies used in immunostaining, immunohistochemistry, western blot and blocking experiments were Fluorescein-Labeled Antibody To Rabbit IgG (H+L) (KPL, 02-15-06), Fluorescein-Labeled Antibody To Mouse IgG (H+L) (KPL, 02-18-06), Cy3 goat anti-mouse(Life Technologies, A10521), Cy3 goat anti-rabbit (Life Technologies, A10520), DAPI (Vector Laboratories, H-1200), 594 donkey anti-sheep IgG (H+L) (Thermo Fisher, A-11016), 488 donkey anti-RAT IgG (H+L) (Abcam, ab150153), IgG-Ms(Sigma, A4416), IgG-Rb (Sigma, A6154).
The antibodies used for FACS were: PE Rat Anti-Mouse CD24 (clone M1/69, BD Biosciences, 553262), FITC Hamster Anti-Rat CD29 (clone Ha2/5, BD Biosciences, 555005), Biotin Rabbit Anti-Mouse TER-119 (clone TER-119, BD Biosciences, 553672), Biotin Rabbit Anti-Mouse CD31 (clone MEC 13.3, BD Biosciences, 553371), Biotin Rat Anti-Mouse CD45R/B220 (clone RA3-6B2, BD Biosciences, 553086), Purified Rat Anti-Mouse CD16/CD32 (clone 2.4G2, BD Biosciences, 553142), Streptavidin-PE-Cy7 (BD Biosciences, 557598), APC Anti-Mouse CD45.2 (clone 104, Biolegend, 109813), FITC Anti-Mouse CD45.2 (clone 104, Biolegend, 109805), PE Anti-Mouse CD45.2 (clone 104, Biolegend, 109807), Pactific BlueTM Anti-Mouse CD45.2 (clone 104, Biolegend, 109819), APC Anti-Mouse/Human CD11b (clone M1/70, Biolegend, 101211), PE Anti-Mouse F4/80 (clone BM8, Biolegend, 123109), APC Anti-Mouse Ly-6G/Ly-6C (Gr-1) (clone RB6-8C5, Biolegend, 108412), FITC Anti-Mouse CD4 (clone RM4-5, Biolegend, 100509), APC Anti-Mouse CD3ε Antibody (clone 145-2C11, Biolegend, 100311), PE Anti-Mouse CD8a (clone 53-6.7, Biolegend, 100707), PE Anti-Mouse CD19 (clone 6D5, Biolegend, 115507), FITC Anti-Mouse/Human CD45R/B220 (clone RA3-6B2, Biolegend, 103205), Pacific Blue™ Anti-Mouse CD11c (clone N418, Biolegend, 117322), APC Anti-Mouse I-A/I-E (MHC II) (clone M5/114.15.2, Biolegend, 107613), CD206 (Abcam, ab64693), Cx3cr1 (Abcam, ab8021), F4/80 (clone Cl:A3-1, Bio-RAD, MCA497G). The secondary antibodies used in FACS were: 488 donkey anti-RAT IgG (H+L) (Abcam, Ab150153), HRP, Goat anti-Rabbit IgG (Sigma, A6154), HRP, Goat anti-RAT IgG (Abbkine, A21040).

Validation

Each antibody has been validated by the companies and by the result of the paper. CD146 (clone EPR3208, Abcam,ab75769) was used for WB, and suitable for mice, which had been validated at Feng J et al, 2023. MCAM (CST, 81701) was used for IF, and suitable for mice, which had been validated by the companies and our study. K14 (clone LL002, Abcam, ab49806) and K18 (clone C-04, Abcam, ab668) were used for IF, and suitable for mice, and validated by Zhao et al., 2020. Ki67 (Abcam, ab15580) were used for IF and IHC, and suitable for mice, and validated by Zhao et al., 2020. CD206 (Abcam, ab64693) was used for IF, and suitable for mice, which had been validated by the companies and our study.Cx3cr1(Abclonal, A2890) was used for IF, and suitable for mice, which had been validated by the companies and our study. α-SMA(clone 1A4, Abcam, ab7817) was used for IF, and suitable for mice, which had been validated by the companies and our study. Wnt5a (Abcam, ab235966) was used for IHC, and suitable for mice, which had been validated by the companies and our study. Wnt5a(Abclonal, A12744) was used for IF, and suitable for mice, which had been validated by the companies and our study. Ryk (Abcepta, AP7677a) was used for IF and IHC, and suitable for mice, which had been validated by the companies and our study. Ryk(R&D Systems, AF4649) was used for IF, and suitable for mice, which had been validated by the companies and our study. InVivoPlus anti-mouse IL4(clone 11B11, BioXcell, BP0045-5MG) and InVivoPlus rat IgG1 isotype control (anti-HRP) (clone HRPN, BioXcell, BP0088-5MG) were blocking antibody, and suitable for mice, which had been validated by the companies and our study. STAT6(clone D3H4, CST, 5397S) was used for WB, and suitable for mice, which had been validated by Woetmann, A. et al. 2003. p-STAT6(CST, 9361S) was used for WB, and suitable for mice, which had been validated by Wei, et. al., 2023. α- tubulin(Sigma, T5168) and Gapdh(Bioworld, AP0063) were used for WB, and suitable for mice, which had been validated by the companies and our study. PE Rat Anti-Mouse CD24, FITC Hamster Anti-Rat CD29, Biotin Rabbit Anti-Mouse TER-119, Biotin Rabbit Anti-Mouse CD31 and Biotin Rat Anti-Mouse CD45R/B220 were used for mice mammary gland epithelial cells by FACS, which had been validated by Zhao et al., 2019. Other antibodies for various immune cells was used for FACS, and suitable for mice, which had been validated by the companies and our study.

Eukaryotic cell lines

Policy information about cell lines and Sex and Gender in Research

Cell line source(s)

HEK293T was purchased from Conservation Genetics CAS Kunming Cell Bank (KCB 200744YJ). RAW264.7 was a gift from Xiaopeng Qi lab, Shandong University.

Authentication

None of the cell line used have been authenticated.

Mycoplasma contamination

All the cell lines are mycoplasma-free.

Commonly misidentified lines (See ICLAC register)	No commonly misidentified cell lines were used in the study.

Animals and other research organisms

Policy information about studies involving animals; ARRIVE guidelines recommended for reporting animal research, and Sex and Gender in Research

Laboratory animals	C57/B6 mice at 8 weeks were purchased from the Experimental Animal Center of the Kunming Institute of Zoology (China). The Mcam floxed mice at 8 weeks were a gift from Xiyun Yan lab, Institute of Biophysics, Chinese Academy of Sciences. The MMTV-Cre (line D) and K14-Cre mice at 8 weeks were obtained from the Jackson Laboratory (USA). The female NOD/SCID mice at 3 weeks were purchased from company of Charles River. The mice were raised in an specific pathogen free (SPF) environment with an ambient temperature of 18-22 °C, a humidity of 50%-60%, and a 12h light-dark cycle.
Wild animals	The study did not involve wild animals.
Reporting on sex	The study did not involve sample collected from the field.
Field-collected samples	The study did not involve sample collected from the field.
Ethics oversight	All animal studies comply with relevant ethical regulations for animal testing and research, and were approved by the Institutional Animal Care. Animals were maintained and studies were carried out in accordance with institutional guidelines.

Note that full information on the approval of the study protocol must also be provided in the manuscript.

Plants

Seed stocks	*Report on the source of all seed stocks or other plant material used. If applicable, state the seed stock centre and catalogue number. If plant specimens were collected from the field, describe the collection location, date and sampling procedures.*
Novel plant genotypes	*Describe the methods by which all novel plant genotypes were produced. This includes those generated by transgenic approaches, gene editing, chemical/radiation-based mutagenesis and hybridization. For transgenic lines, describe the transformation method, the number of independent lines analyzed and the generation upon which experiments were performed. For gene-edited lines, describe the editor used, the endogenous sequence targeted for editing, the targeting guide RNA sequence (if applicable) and how the editor was applied.*
Authentication	*Describe any authentication procedures for each seed stock used or novel genotype generated. Describe any experiments used to assess the effect of a mutation and, where applicable, how potential secondary effects (e.g. second site T-DNA insertions, mosiacism, off-target gene editing) were examined.*

Flow Cytometry

Plots

Confirm that:

☒ The axis labels state the marker and fluorochrome used (e.g. CD4-FITC).

☒ The axis scales are clearly visible. Include numbers along axes only for bottom left plot of group (a 'group' is an analysis of identical markers).

☒ All plots are contour plots with outliers or pseudocolor plots.

☒ A numerical value for number of cells or percentage (with statistics) is provided.

Methodology

Sample preparation	Mammary glands from 8–10-week-old C57/B6 female mice were minced and digested in Dulbecco's Modified Eagle's Medium/Nutrient Mixture F-12 (DMEM/F12) containing 5% fetal bovine serum (FBS), 1% penicillin-streptomycin, 300 U/ml collagenase I (Sigma, C0130), and 100 U/ml hyaluronidase (Sigma, H3506) for 1–2 h at 37 °C. Single-cell suspension was obtained by sequential incubation with 0.25% trypsin EDTA for 5 min and 5 mg/ml dispase (Sigma, D4693) containing 0.1 mg/ml Dnase I (Roche, 11248932001) for 5 min at 37 °C with gentle shaking. Finally, red blood cells were removed with 0.8% NH_4Cl, and the cell suspension was then filtered through a 40-µm cell strainer.
Instrument	Beckman Moflo Asrios EQs, Beckman
Software	The software used to collect and analyze the flow cytometry data is FlowJo 10.8.1.
Cell population abundance	The abundance of each population mentioned in this paper has been shown in the figures.

Gating strategy

The gating strategy has been presented and described in the paper.

☒ Tick this box to confirm that a figure exemplifying the gating strategy is provided in the Supplementary Information.
